# A New Approach to Handle Missing Covariate Data in Twin Research: With an Application to Educational Achievement Data

**DOI:** 10.1007/s10519-015-9771-1

**Published:** 2015-12-19

**Authors:** Inga Schwabe, Dorret I. Boomsma, Eveline L. de Zeeuw, Stéphanie M. van den Berg

**Affiliations:** Department of Research Methodology, Measurement, and Data Analysis, University of Twente, Drienerlolaan 5, 7522 NB Enschede, Netherlands; Department of Biological Psychology, VU University Amsterdam, Van der Boechorststraat 1, 1081 BT Amsterdam, Netherlands

**Keywords:** Twin studies, Covariates, Missing data, Educational achievement

## Abstract

The often-used ACE model which decomposes phenotypic variance into additive genetic (A), common-environmental (C) and unique-environmental (E) parts can be extended to include covariates. Collection of these variables however often leads to a large amount of missing data, for example when self-reports (e.g. questionnaires) are not fully completed. The usual approach to handle missing covariate data in twin research results in reduced power to detect statistical effects, as only phenotypic and covariate data of individual twins with complete data can be used. Here we present a *full information* approach to handle missing covariate data that makes it possible to use *all* available data. A simulation study shows that, independent of missingness scenario, number of covariates or amount of missingness, the full information approach is more powerful than the usual approach. To illustrate the new method, we applied it to test scores on a Dutch national school achievement test (*Eindtoets Basisonderwijs*) in the final grade of primary school of 990 twin pairs. The effects of school-aggregated measures (e.g. school denomination, pedagogical philosophy, school size) and the effect of the sex of a twin on these test scores were tested. None of the covariates had a significant effect on individual differences in test scores.

## Introduction

**Fig. 1:**
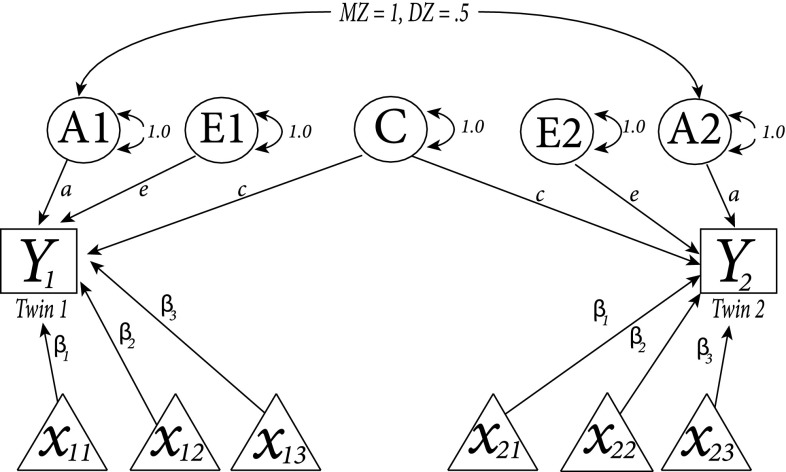
Structural equation model (SEM) for a basic univariate twin analysis (ACE model) extended with three covariates (denoted as $x_{11}, x_{12}$ and $x_{13}$ for the first and as $x_{21}$, $x_{22}$ and $x_{23}$ for the second twin of a family). *Y* denotes the phenotypic values of the first (*Y*1) and second (*Y*2) twin and *A* refers to additive genetic influences for the first (*A*1) and second (*A*2) twin, which are correlated 0.5 in dizygotic twins and 1 in monozygotic twins. *E*1 and *E*2 denote unique-environmental influences of the first and second twin respectively and are assumed to be uncorrelated. *C*, common-environmental influences, are the same for all family members. Double-headed arrows denote (co-)variances. The path coefficients, $\beta _{1}$, $\beta _{2}$, $\beta _{3}$, *a*, *c* and *e* represent regression coefficients that express the estimated effect of the respective influences

In the genomics era, twin studies remain useful to estimate the relative importance of genetic and environmental influences on individual differences. In the often-used ACE model, the total variance of a trait (e.g. mathematical ability) is decomposed into components due to additive genetic (A) influences, common-environmental (C) influences that are shared by family members and unique-environmental (E) influences (Jinks and Fulker 1970). This model can be extended to include covariates. Figure 1 is an example of the structural equation model (SEM) for a basic univariate twin analysis extended with three covariates (denoted as $x_{11}, x_{12}$ and $x_{13}$ for the first twin and $x_{21}, x_{22}$ and $x_{23}$) for the second twin of one family). The path coefficients $\beta _{1}, \beta _{2}$ and $\beta _{3}$ represent regression coefficients that express the estimated effect of the respective covariate. This model implies that the ACE variance decomposition takes place on the residuals of the phenotypic scores, after the effects of the covariates have been partialled out.

### Missing covariate data

The collection of covariate data however often leads to a high amount of missingness. For example, when self-reports (e.g. questionnaires) are used to gather information on the environment of a family or an individual twin, they are often not fully completed (e.g. the last items are skipped) or items on sensitive topics (e.g. alcohol or drug use) are not answered. Likewise, the linkage of two datasets may lead to missing data. A twin researcher might want to link twin data from a twin registry to data from the same twins from another (external) source. For example, an environmental variable such as the socio-economic status of a neighbourhood might not be available in the twin registry, but there is a publicly available dataset from a governmental or local organisation which includes the desired variable. For the linking of the two datasets, usually, a common identifier such as the name or address of a family or individual twin can be used. However, this potentially leads to a lot of missing data, for example when entities cannot be (uniquely) linked to the common identifier, as may be the case due to differences in record shape or choice of identification variables.

We can distinguish between three different mechanisms that describe relationships between measured variables and the probability of missing data (Rubin 1976; Little and Rubin 2002). Data are said to be *missing completely at random* (MCAR) when the probability that a value is missing is unrelated to both observed and unobserved data. For example, a respondent might flip a coin to decide whether to answer a questionnaire item or not. Note that this is a rather strong assumption. A weaker assumption is that covariates are *missing at random* (MAR), that is, the probability that a covariate value is missing is unrelated to its unobserved value, after controlling for other variables in the analysis. For example, female twins might be more likely to not give information on their income, but this might be unrelated to the amount of income once one controls for gender. Lastly, a covariate value can be *missing not at random* (MNAR), that is, the probability that it is missing is related to its unobserved value. In this case, for example, twins with a lower income might be more or less likely to reveal this information.

When there is (partly) missing data, complete-cases analysis (also referred to as listwise deletion) can be used, meaning that only twin pairs with complete data (e.g. data of twin pairs with known values for all covariates) enter the analysis. This certainly leads to reduced statistical power, but might also introduce bias or affect the representativeness of the results (Allison 2001). Using OpenMx (Boker et al. 2011), a SEM program often used to fit twin models, twin researchers usually apply a strategy that minimizes the loss of information by excluding phenotypic data of *individual* twins with at least one missing covariate value. So, when an individual twin has a missing covariate value, the phenotypic (and covariate) data of his or her co-twin can still be used for statistical inference (provided that the co-twin does not have any missing data). This results in twin-wise rather than twin pair-wise deletion of incomplete cases.

In this paper, we present a *full information* approach to handle missing covariate data. The new approach involves including covariates in the expected covariance matrix. While in the usual approach, the phenotypic and covariate values of a twin with (at least) one missing covariate value are completely ignored, the full information approach models *all* data that is observed - including observed phenotypic data as well as observed covariate data. The new approach will be described in more detail in the following.

### Full information approach

In the traditional univariate ACE model, the phenotypic variance is decomposed into variance due to additive genetic influences, $\sigma ^2_A$, variance explained by common-environmental influences, $\sigma ^2_C$, and variance due to unique-environmental influences, $\sigma ^2_E$. Conditioning on the covariate data, phenotypic data are assumed to be multivariate normally distributed:

1 $$\begin{aligned} \begin{array}{ll} y_{i1} \\ y_{i2} \end{array}\ {\mid }{ \begin{array}{ll} \varvec{x}_{i1} \\ \varvec{x}_{i2} \end{array}} \sim MVN \Bigg ( \begin{pmatrix}\mu_y \\ \mu_y \end{pmatrix} + \begin{pmatrix} \varvec{x}_{i1}^T \\ \varvec{x}_{i2}^T \end{pmatrix} \varvec{\beta } , {\mathrm {\Sigma }}_{ACE} \Bigg ) \end{aligned}$$

where

2 $$\begin{aligned} {\mathrm {\Sigma }}_{ACE} = \begin{bmatrix} \sigma ^2_A + \sigma ^2_C + \sigma ^2_E&\rho \sigma ^2_A + \sigma ^2_C \\ \rho \sigma ^2_A + \sigma ^2_C&\sigma ^2_A + \sigma ^2_C + \sigma ^2_E \end{bmatrix} \end{aligned}$$

and $\mu _y$ refers to the phenotypic mean. $y_{i1}$ denotes the phenotypic value of the first twin of family *i* and $y_{i2}$ represents the phenotypic value of the second twin. $\varvec{x}_{i1}$ and $\varvec{x}_{i2}$ are covariate data vectors that include the values of the covariates of the first and second twin respectively and the vector $\varvec{\beta }$ consists of the regression coefficients of the covariates. ${\mathrm {\Sigma _{ACE}}}$ refers to total phenotypic covariance and $\rho$ is the correlation between the twins’ additive polygenic factors, which is unity in monozygotic (MZ) twins and $\frac{1}{2}$ in dizygotic (DZ) twins. The phenotypic variance decomposition takes place after the effects of the covariates have been partialled out, but other than that the covariate data are not part of the covariance model.

Here, we propose to model the covariance between *all* observed variables - consisting of phenotypic data but also covariate data. In twin data, it is reasonable to assume not only covariance among the covariates of one twin (e.g. correlations between covariates), but also covariance among the values of the covariates of one twin and the covariates of the co-twin. To incorporate this dependence structure into the biometric model we decompose the covariance structure of the values on the covariates of both twins into covariance shared by twins from the same pair and non-shared twin covariance. Covariate data were then assumed to be multivariate normally distributed:

3 $$\begin{aligned} \begin{array}{ll} \varvec{x}_{i1} \\ \varvec{x}_{i2} \end{array} \sim MVN \Bigg ( \begin{pmatrix} \varvec{\mu }_x \\ \varvec{\mu }_x \end{pmatrix}, {\mathrm {\Sigma _{cov}}} \Bigg )\ \end{aligned}$$

where $\varvec{\mu }_x$ is a vector that contains of the means of the covariates and

4 $$\sum\nolimits_{{\rm{COV}}} = \left[ {\begin{array}{ll} {\sum\nolimits_{{\rm{twin}}1}}&{\sum\nolimits_{\rm{b}} }\\ {\sum\nolimits_{\rm{b}}}&{\sum\nolimits_{{\rm{twin}}2} } \end{array}} \right] = \left[ {\begin{array}{ll} {\sum\nolimits_{\rm{W}} { + \sum\nolimits_{\rm{b}} } }&{\sum\nolimits_{\rm{b}}}\\ {\sum\nolimits_{\rm{b}}}&{\sum\nolimits_{\rm{W}} { + \sum\nolimits_{\rm{b}} } }\end{array}} \right]$$

${\mathrm {\Sigma _{cov}}}$ denotes total covariate covariance. ${\mathrm {\Sigma _b}}$ denotes between twin pair variance and ${\mathrm {\Sigma _w}}$ within twin pair variance. Thus, the covariance matrix for covariates of an individual twin is decomposed into covariance shared with the co-twin, ${\mathrm {\Sigma _b}}$, and covariance not shared with the co-twin, ${\mathrm {\Sigma _w}}$.

**Fig. 2:**
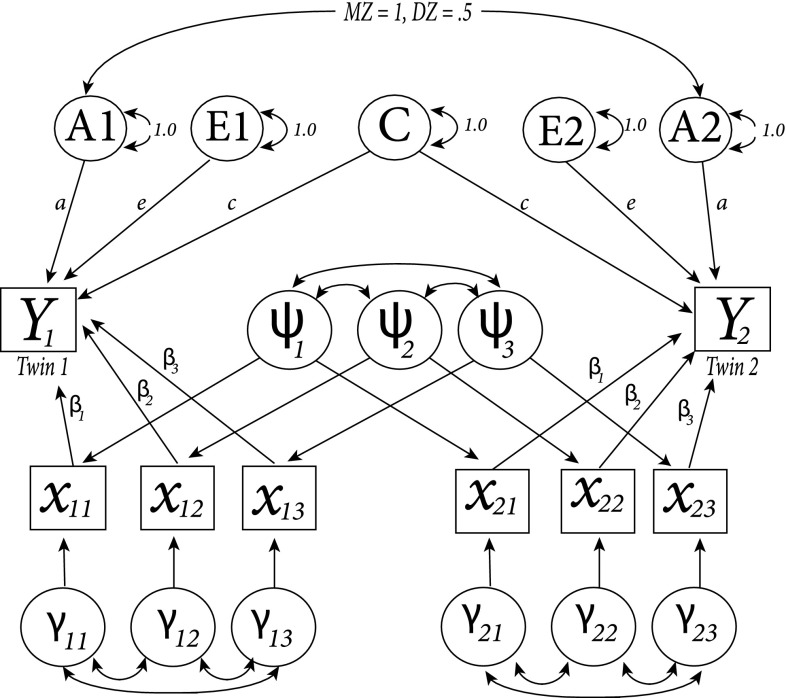
Structural equation model (SEM) of the full information approach. Answers to three different covariates are displayed for the first ($x_{11}, x_{12}, x_{13}$) and second twin ($x_{21}, x_{22}, x_{23}$) of one family. $\psi$ is a latent variable that is estimated for every covariate ($\psi _1, \psi _2, \psi _3$) and models covariance within families. The different latent variables for the first ($\gamma _{11}, \gamma _{12}$ and $\gamma _{13}$) and second ($\gamma _{21}, \gamma _{22}$ and $\gamma _{23}$) twin model within twin covariance

By including covariate data in the expected covariance matrix, the joint distribution of phenotypes and covariates, $(y_{i1}, y_{i2}, \varvec{x}_{i1},\varvec{x}_{i2})^T$, is multivariate normal with the following covariance structure:

5 $$\begin{aligned} {\mathrm {\Sigma }} = \left[ \begin{array}{ll} ACE + \varvec{\beta }^T({\mathrm {\Sigma _w}}+{\mathrm {\Sigma _b}})\varvec{\beta }\quad \\ AC+ \varvec{\beta }^T{\mathrm {\Sigma _b}}\varvec{\beta } \\ ({\mathrm {\Sigma _w}}+{\mathrm {\Sigma _b}})\varvec{\beta } \\ {\mathrm {\Sigma _b}}\varvec{\beta } \end{array} \begin{array}{ll} AC+\varvec{\beta }^T {\mathrm {\Sigma _b}} \varvec{\beta } \\ ACE+\varvec{\beta }^T ({\mathrm {\Sigma _w}}+{\mathrm {\Sigma _b}})\varvec{\beta } \quad \\ {\mathrm {\Sigma _b}} \varvec{\beta } \\ ({\mathrm {\Sigma _w}}+{\mathrm {\Sigma _b}})\varvec{\beta } \end{array} \begin{array}{ll} \varvec{\beta }^T({\mathrm {\Sigma _w}}+{\mathrm {\Sigma _b}}) \quad \\ \varvec{\beta }^T {\mathrm {\Sigma _b}} \\ {\mathrm {\Sigma _w}}+{\mathrm {\Sigma _b}} \\ {\mathrm {\Sigma _b}} \end{array} \begin{array}{ll} \varvec{\beta }^T {\mathrm {\Sigma _b}}\\ \varvec{\beta }^T({\mathrm {\Sigma _w}}+{\mathrm {\Sigma _b}}) \\ \mathrm {\Sigma _b} \\ \mathrm {\Sigma _w} + \mathrm {\Sigma _b} \end{array}\right] \end{aligned}$$

where phenotypic variances are represented on the first two elements of the diagonal and covariate data variances on the remaining elements of the diagonal. Cross-phenotypic and cross-covariates covariances and within twin covariate covariances are contained on the off-diagonal elements. *ACE* denotes $\sigma ^2_A + \sigma ^2_C + \sigma ^2_E$, *AC* refers to $\rho \sigma ^2_A + \sigma ^2_C$ and $\varvec{\beta }$ is a vector that includes the regression coefficients of the covariates. A graphical representation of this model, including ACE decomposition and the model for covariate data, can be found in Fig. 2 (SEM notation). In the example, answers to three different covariates are modelled for the first ($x_{11}, x_{12}$ and $x_{13}$) and second ($x_{21}, x_{22}$ and $x_{23}$) twin of one family. To model between twin pair variance (i.e., ${\mathrm {\Sigma _b}}$, covariance between the values of the first and second twin on the same covariate but also cross-covariance), we model latent variables for every covariate, $\psi _1, \psi _2$ and $\psi _3$. To model within twin pair variance (i.e., ${\mathrm {\Sigma _w}}$), we use different latent variables for the first ($\gamma _{11}, \gamma _{12}$ and $\gamma _{13}$) and second ($\gamma _{21}, \gamma _{22}$ and $\gamma _{23}$) twin.

### Benefits of the new approach

In the usual approach, the phenotypic score as well as the covariate data of a twin with (at least) one missing value is not used for statistical inference. Adopting the full information approach, all observed data (including phenotypic scores) can be used. This fact alone makes the full information approach more powerful.

Furthermore, the usual approach may result in biased estimates when covariate data were missing not completely at random. Imagine for example that the probability that a covariate value is missing depends on the phenotypic value of an individual twin. For example, twins with a high score on a depression assessment are less likely to give information on their income. Using the usual approach, the phenotypic values of these twins do not enter the analysis - therefore, phenotypic variance is underestimated, which might lead to biased estimates of variance components and under- or overestimation of heritability.

The third advantage is that, by modelling the relationships between data that are unobserved and data that are observed, information on model parameters can be statistically borrowed by information on data that are observed and that is correlated with unobserved data - a principle that is often referred to as *borrowing strength*. Borrowing strength means that the inference of a parameter of interest or unobserved data point can be improved by borrowing from information on other related data also included in the model. For example, imagine that we have measured one environmental covariate, separately for the first and second twin of every family. In one of the families, the covariate value of the second twin is known whereas the value for the first twin is missing. Based on covariance between the observed values, our model can then borrow information from the covariate value of the co-twin but also from the phenotypic value of the twin with missing value and the phenotypic value of his or her co-twin, which leads to lower standard errors. Note that this is especially true when the data are highly correlated, for example with high twin correlations for a covariate, high correlations among the covariates, or when there is a strong relationship between phenotypic and covariate data.

In a simulation study, it is shown that the full information approach is more powerful than the usual approach, independent of missingness scenario, number of covariates and amount of missingness. To illustrate the new approach, it is applied to test scores on a Dutch national school achievement test. Syntax to apply the full information approach using the R package OpenMx (Boker et al. 2011) can be found in the Appendix.

## Simulation study

In order to show that the full information approach retrieves parameters reliably and is more powerful than the usual approach, a simulation study was conducted with a fixed number of twin pairs and different number of covariates (two, three, four and five) and percent of missing observations (2, 6 and 10 %). In each combination of these conditions, 1000 datasets were generated consisting of 280 MZ (28 % of all pairs) and 720 DZ pairs (72 % of all pairs). This ratio reflects the usual ratio of MZ and DZ twin pairs in European twin registers. The amount of missing observations for the different conditions (2, 6 and 10 %) was based on the total number of covariate answers (e.g., in case of five covariates: five $\times$ 2000 individual twins = 10,000). In all conditions, additive genetic variance was assumed 0.5, common-environmental variance was set to 0.3 and unique-environmental variance was assumed 0.2. The data were simulated with a phenotypic population mean of zero for all twins ($\mu _y = 0$). In every condition, regression coefficients, $\varvec{\beta }$, were chosen such that covariates explained 39 % of total phenotypic variance, leading to a total variance of 1.64. A multivariate normal distribution was used to simulate the covariate data. The expectation of the multivariate distribution was set to zero ($\varvec{\mu }_x$ = 0) and the covariance matrix was based on ${\mathrm {\Sigma _w}}$ and ${\mathrm {\Sigma _b}}$. The same values were used for the diagonals and off-diagonals of ${\mathrm {\Sigma _w}}$ and ${\mathrm {\Sigma _b}}$ in every condition. For example for five covariates, ${\mathrm {\Sigma _w}}$ was equal to $$\left( {\begin{matrix} 1 &{} .1 &{} .1 &{} .1 &{} .1 \\ .1 &{} 1 &{} .1 &{} .1 &{} .1 \\ .1 &{} .1 &{} 1 &{} .1 &{} .1 \\ .1 &{} .1 &{} .1 &{} 1 &{} .1 \\ .1 &{} .1 &{} .1 &{} .1 &{} 1 \\ \end{matrix}} \right)$$ and ${\mathrm {\Sigma _b}}$ was equal to $$\left( {\begin{matrix} 1 &{} .5 &{} .5 &{} .5 &{} .5 \\ .5 &{} 1 &{} .5 &{} .5 &{} .5 \\ .5 &{} .5 &{} 1 &{} .5 &{} .5 \\ .5 &{} .5 &{} .5 &{} 1 &{} .5 \\ .5 &{} .5 &{} .5 &{} .5 &{} 1 \\ \end{matrix}} \right)$$. This led to following covariance matrix:

6 $$\begin{aligned} {\mathrm {\Sigma _{tot}}} = \begin{bmatrix} 2&.6&.6&.6&.6 \,&1&.5&.5&.5&.5 \\ .6&2&.6&.6&.6 \,&.5&1&.5&.5&.5 \\ .6&.6&2&.6&.6 \,&.5&.5&1&.5&.5 \\ .6&.6&.6&2&.6 \,&.5&.5&.5&1&.5 \\ .6&.6&.6&.6&2 \,&.5&.5&.5&.5&1 \\ \\ 1&.5&.5&.5&.5 \,&2&.6&.6&.6&.6 \\ .5&1&.5&.5&.5 \,&.6&2&.6&.6&.6 \\ .5&.5&1&.5&.5 \,&.6&.6&2&.6&.6 \\ .5&.5&.5&1&.5 \,&.6&.6&.6&2&.6 \\ .5&.5&.5&.5&1 \,&.6&.6&.6&.6&2 \\ \end{bmatrix} \end{aligned}$$

The pattern of the missingness was generated under three different scenarios. In the first setting, covariate data were simulated to be missing completely at random (MCAR). That is, every covariate value had the same probability of being missing, independent of unobserved or observed data. In the second scenario, it was assumed that the data were missing at random (MAR). Here, the probability that a covariate value was missing was dependent on the (observed) phenotypic value of an individual twin. We modelled the probability of missingness for every covariate $x_{ijk}$ as a logistic function of the respective phenotypic value of every individual twin *j* from family *i*:

7 $$\begin{aligned} p(x_{ijk} \; {\text{is missing}}) = \frac{1}{1 + {\text{exp}}(2 + 1.7 \, y_{ij})} \end{aligned}$$

The resulting probabilities were then used in the R in-built function *sample()* to control the overall proportion of missing values. By using Eq. 7 to model missingness, the probability that a covariate value was missing was higher with decreasing phenotypic value. In the last scenario, covariate data were assumed to be missing not at random (MNAR). Here, the probability that a covariate value was missing was dependent on its observed (simulated) value. As the range of phenotypic values was similar to the range of covariates values, the same logistic function was used as in the MAR scenario, but the probability was dependent on the observed value of the covariate (i.e. $p(x_{ijk} \; {\text{is missing}}) = \frac{1}{1 + {\text{exp}}(2 + 1.7 \, x_{ijk})}$). As in the MAR setting, the resulting probabilities were used in the R in-built function *sample()* to control the overall proportion of missing values.

In every scenario, the remaining data were analysed using 1) the usual approach and 2) the full information approach. For the simulations, the software package R (R Development Core Team 2008) was used. The models were fit using the R package OpenMx (Boker et al. 2011). The point estimates of the variance components and regression coefficients were determined as were their standard errors. Furthermore, narrow-sense heritability, $h^2$, was determined, which we defined here as $\frac{\sigma ^2_A}{\sigma ^2_P}$, where $\sigma ^2_P = \sigma ^2_A + \sigma ^2_C + \sigma ^2_E$.

### Results

As estimates of regression coefficients were close to their true values and very similar for both approaches under all conditions, results are not displayed here but can be obtained from the first author.

**Fig. 3:**
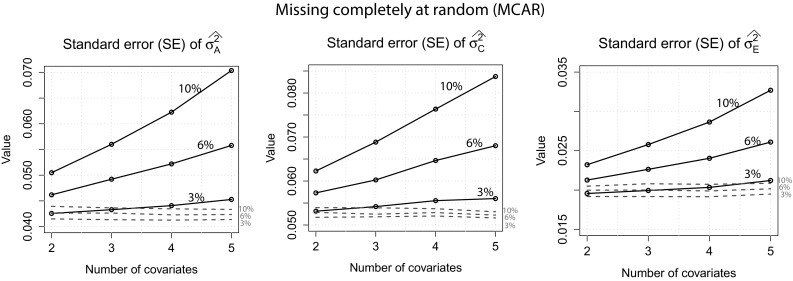
*MCAR*: Standard errors for $\sigma ^2_A$, $\sigma ^2_C$ and $\sigma ^2_E$ of both approaches when 2, 6 and 10 % of the covariate data were missing. *Dotted lines* full information approach

MCAR: Standard errors for $\sigma ^2_A$, $\sigma ^2_C$ and $\sigma ^2_E$ can be found in Fig. 3. The standard errors were generally lower when the full information approach was used compared to the usual approach. Furthermore, while standard errors were very similar under different amounts of missingness and number of covariates when the full information approach was applied, they increased with increasing number of covariates when the usual approach was used. This effect was the largest for the 10 % missingness condition. Compared to the other variance components, standard errors of $\sigma ^2_E$ were, in general, small and only increased slightly with increasing number of covariates when the usual approach was used. For both approaches, estimates of $\sigma ^2_A$, $\sigma ^2_C$, $\sigma ^2_E$ and $h^2$ were all very close to their true values and are therefore not displayed here.

**Fig. 4:**
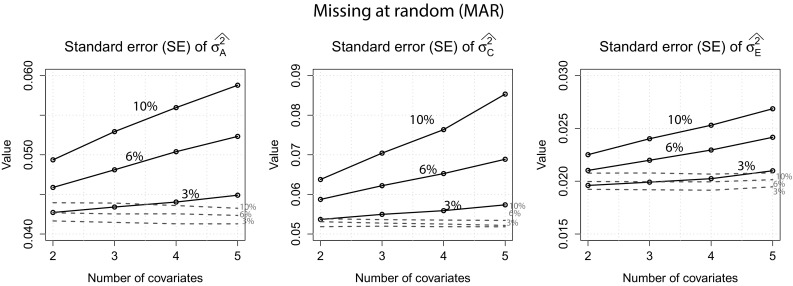
*MAR*: Standard errors for $\sigma ^2_A$, $\sigma ^2_C$ and $\sigma ^2_E$ of both approaches when 2, 6 and 10 % of the covariate data were missing. *Dotted lines* full information approach

MAR: The standard errors for $\sigma ^2_A$, $\sigma ^2_C$ and $\sigma ^2_E$ can be found in Fig. 4.

**Fig. 5:**
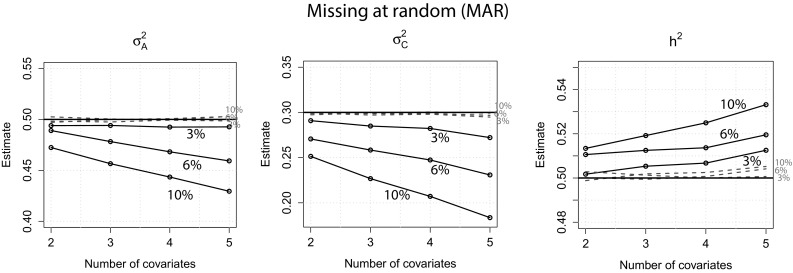
*MAR*: Estimates of $\sigma ^2_A$, $\sigma ^2_C$ and $h^2$ of both approaches when 2, 6 and 10 % of the covariate data were missing. *Dotted lines* full information approach. The *horizontal lines* denote the true values of $\sigma ^2_A$, $\sigma ^2_C$ and $h^2$

The same pattern as for the MCAR condition can be observed: Standard errors of the full information approach were generally lower than the standard errors obtained with the usual approach. Furthermore, while the standard errors of the usual approach increased with increasing number of covariates, standard errors of the full information approach were very similar across all misisngness conditions. As in the first scenario, standard errors for $\sigma ^2_E$ were generally low and increased only slightly with increasing number of covariates when the usual approach was used. Figure 5 displays the estimates of $\sigma ^2_A$, $\sigma ^2_C$ and $h^2$ for both approaches.

It can be seen that the variance components estimates of the full information approach were all very close to their true values, independent of amount of missingness and missingness scenario. Using the usual approach, estimates were close to their true values in the 3 % missingness condition, but variance components were underestimated when the amount of missignness increased. This bias further increased with increasing number of covariates. Furthermore, the bias was generally more severe for estimates of $\sigma ^2_C$ than for estimates of $\sigma ^2_A$. This is also reflected in the estimates for $h^2$. Estimates of heritability were overestimated, which systematically increased with increasing number of covariates. However, we can also see that this effect was negligible for the 3 and 6 % missingness condition. Estimates of $\sigma ^2_E$ were unbiased for the full information approach as well as the usual approach and are therefore not displayed.

**Fig. 6:**
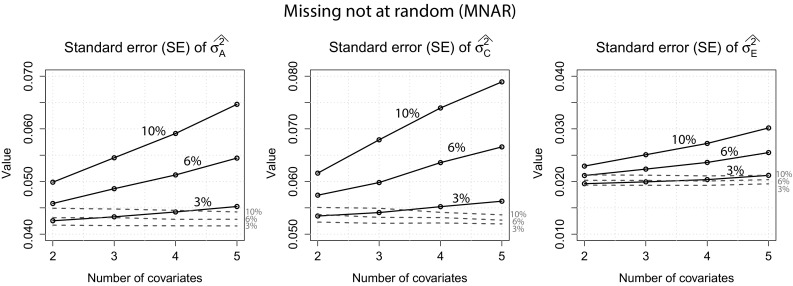
*MNAR*: Standard errors for $\sigma ^2_A$, $\sigma ^2_C$ and $\sigma ^2_E$ of both approaches when 2, 6 and 10 % of the covariate data were missing. *Dotted lines* full information approach

MNAR: Figure 6 shows the standard errors for both approaches for all variance components. Again, we can observe the same pattern: the full information approach had lower standard errors which were very similar under different conditions while standard errors increased with increasing number of covariates when the usual approach was used. Similar to the MCAR and MAR scenario, the standard errors of $\sigma ^2_E$ only increased slightly with increasing number of covariates and were generally low also when the usual approach was used. Estimates of $\sigma ^2_A$, $\sigma ^2_C$ and $\sigma ^2_E$ were all very close to their true values for both approaches and are therefore not displayed here. Using the full information approach, there was a negligible bias in the 10 % missingness condition with estimates of $\sigma ^2_E$ closer to 0.21 instead of the true value 0.20.

## Application

To illustrate the full information approach, we applied it to test scores on a Dutch national school achievement test in the final grade of primary school. The effect of school-aggregated measures (e.g. school denomination, pedagogical philosophy, school size) and the effect of the sex of a twin on these test scores was tested. These covariates were a mix of continuous and categorical variables. Therefore, due to its flexibility, it was chosen to use a Bayesian parametrization of the model for this application. In Bayesian analysis, statistical inference is based on the joint *posterior density* of the model parameters, which is proportional to the product of a prior probability distribution and the likelihood function (for a general introduction to Bayesian statistics see e.g. Bolstad 2007 and for Bayesian analysis of twin models see e.g. Eaves and Erkanli 2003 or van den Berg et al. 2006). A prior probability distribution represents information about an uncertain parameter before any data have been observed. In this application, uninformative prior distributions were chosen. That is, they expressed only vague information about the parameters of our model and therefore, posterior point estimates presented here are close to maximum likelihood estimates as would be obtained by using for example OpenMx (Boker et al. 2011).

### Sample

The sample of this study originated in the Netherlands Twin Register (NTR, Boomsma et al. 2002), which includes approximately 40 per cent of all multiple births in the Netherlands. If parents give their consent, teachers of the children are approached with a survey when the twins are 7, 9 and 12 years old. In 2000, the NTR started collecting the results of a national test of educational achievement (*Eindtoets Basisonderwijs*) from the parents of all 12-year old twins. The *Eindtoets Basisonderwijs* test is yearly administered in the final grade of primary school.

The present study analyzed data of 12-year-old twins from birth cohorts 1997-2000 to determine the importance of measured covariates for individual differences in *Eindtoets Basisonderwijs* test scores. The sample included data of children from 990 twin pairs, consisting of 340 MZ twin pairs and 650 DZ twin pairs. Of the MZ twin pairs, 175 pairs were male and 165 female. 159 of the DZ twin pairs were male, 167 female and 324 twin pairs were of opposite sex. For 120 individual twins, the score on the *Eindtoets Basisonderwijs* test was unknown. The reason that the score was missing was either that the child had not reached final grade yet (N twins = 66), the child was attending special education (N twins = 33), a different test was used at the school the twin was attending (N twins = 6), the child did not attend the test (N twins = 2) or the reason was unknown (N twins = 23).

### Measures

The items on the teacher report form that were used in this paper are: The name, postal code, denomination and pedagogical philosophy of the school. The reported names and postal codes were used to link the twin data from the NTR with school-aggregated environmental measures obtained from external sources such as official authorities. This was only done with data for twins for which parents had given written permission to link databases. Reported denomination and pedagogical philosophy of a school on the teacher report were used to complement the retrieved data. The *Eindtoets Basisonderwijs* test consists of 290 multiple choice items in four different subjects (language, world studies [optional], arithmetic and study skills). We used the total score on the *Eindtoets Basisonderwijs* test, a standardized measure that ranges from 500 to 550. As administration of the questions concerning world studies is optional, they were not included in the total score. Information on the denomination of a specific school was retrieved from the Dutch ministry of education (Dienst Uitvoering Onderwijs, DUO). This information was supplemented with information available from answers of the teachers on the teacher report form. The variable was measured in seven categories: *Collaboration of Protestant-Christian and Roman Catholic*, *Protestant-Christian*, *Reformed*, *Reformed liberated*, *Roman Catholic*, *Special* and *State* schools. Information on pedagogical philosophy was retrieved online from a database that provides basic information about Dutch primary education schools (http://www.scholenopdekaart.nl). Again, this information was supplemented with information available from answers on the teacher report form. The variable was categorized into five different categories: *Regular education*, *Dalton plan education*, *Jenaplan edcation*, *Montessori education*, *Specialised regular education* and *Specialised education*. Data on school size, measured in 2011, were retrieved from the Dutch ministry of education (Dienst Uitvoering Onderwijs, DUO). The data were linked to the postal codes of the schools, retrieved from the teacher report form. An overview of all covariates that were used in this paper can be found in Table 1.

**Table 1:** Overview of covariates for educational achievement (*Eindtoets Basisonderwijs* test scores) that were used in the application.

	*N*
*Sex*	
Boy	992 (50.10 %)
Girl	988 (49.90 %)
*School size*	1447 (73.08 %)
Missing	533 (26.92 %)
*Pedagogical philosophy*	
Regular education	1467 (74.09 %)
Dalton	41 (2.10 %)
Jenaplan	12 (0.61 %)
Montessori	21 (1.06 %)
Specialised regular education	16 (0.81 %)
Specialised education	6 (0.30 %)
Missing	417 (21.10 %)
*Denomination of school*	
Protestant-Christian (PC)	332 (16.77 %)
Reformed	22 (1.11 %)
Reformed liberated	8 (0.40 %)
Roman-Catholic (RC)	606 (30.61 %)
Collaboration of PC & RC	10 (0.51 %)
Special	59 (2.98 %)
State	461 (23.28 %)
Missing	482 (24.34 %)

*N* = total number of individual twins

### Analysis

The analysis was done in the Markov chain Monte Carlo (MCMC) sampling program JAGS (Plummer 2003). R (R Development Core Team 2008) was used for further data handling and as an interface from R to JAGS, the rjags package (Plummer 2013) was used.

Prior to the analysis, $c - 1$ dummy variables were created for the categorical variables *Sex*, *School denomination* and *Pedagogical philosophy* with *c* being the number of categories and the largest category serving as the reference group. For these covariates, the distribution in Eq. 3 was used to model liabilities. The built-in function *step()* of JAGS was then used to create a Boolean variable $V = \text {step}(x_{ijk} - t)$ that equals one if $(x_{ijk} - t) \ge 0$ and equals zero if $(x_{ijk} - t) < 0$ where *t* is a threshold that was fixed to zero for identification purposes. The phenotypic variable (*Eindtoets Basisonderwijs* test scores) as well as the numeric covariate *School size* were standardized to have an expected value of zero and a variance of one. The missing *Eindtoets Basisonderwijs* test scores (N twins = 120) were assumed missing at random.

The mean and standard deviation of the posterior distribution were calculated for each parameter as was the 95 % highest posterior density (HPD, see e.g. Box and Tiao 1992) interval for variance components and the 99.6 % HPD interval for covariates. The HPD can be interpreted as the Bayesian analog of a confidence interval (CI). When the HPD does not contain zero, the influence of a parameter can be regarded as significant.

### Results

The posterior means for the variance components $\sigma ^2_A$, $\sigma ^2_C$ and $\sigma ^2_E$ as well as the estimated heritability ($h^2$, defined as $\frac{\sigma ^2_A}{\sigma ^2_P}$, where $\sigma ^2_P = \sigma ^2_A + \sigma ^2_C + \sigma ^2_E$) for the fitted model are displayed in Table 2. The results suggest that the largest part of the variance could be explained by genetic influences, resulting in a relatively high estimate for heritability. A substantial part of the phenotypic variance could be explained by unique-environmental influences and a small part by common-environmental influences.

**Table 2:** Educational achievement (*Eindtoets Basisonderwijs* test scores): Posterior means (SD) of the variance components

	Posterior mean (SD)	HPD
$\sigma ^2_A$	0.66 (0.05)	[0.57;0.76]
$\sigma ^2_C$	0.16 (0.04)	[0.09;0.24]
$\sigma ^2_E$	0.20 (0.02)	[0.17;0.23]
$h^2$	0.64 (0.04)	[0.56;0.72]

HPD refers to the 95 % highest posterior density interval

The posterior means and HPD intervals of the regression coefficients are displayed in Table 3. There was no covariate that had a significant effect on individual differences in *Eindtoets Basisonderwijs* test scores.

**Table 3:** Educational achievement (*Eindtoets Basisonderwijs* test scores): Regression coefficients for the estimated model

	Sex	School denomination						School size	Pedagogical philosophy				
		Collaboration PC & RC	Protestant-Christian	Reformed	Reformed liberal	Special	State		Dalton	Jenaplan	Montessori	Specialised regular	Specialised
Posterior mean	-0.11 (0.04)	-0.03 (0.14)	0.02 (0.06)	-0.22 (0.13)	-0.11 (0.13)	0.21 (0.11)	0.07 (0.06)	0.04 (0.03)	-0.09 (0.14)	-0.20 (0.16)	0.04 (0.15)	0.27 (0.16)	0.02 (0.16)
HPD	[-0.23;0.00]	[-0.44;0.36]	[-0.17;0.19]	[-0.58;0.12]	[-0.49;0.27]	[-0.12;0.50]	[-0.10;0.23]	[-0.07;0.13]	[-0.49;0.30]	[-0.66;0.24]	[-0.38;0.47]	[-0.18;0.71]	[-0.46;0.48]

For the covariates Sex, School denomination and Pedagogical philosophy, dummy variables were used. The reference categories are: *Female*, *Roman-Catholic* and *Regular education*. HPD refers to the *99.6 % highest posterior density interval*

## Discussion

The often-used ACE model can be extended to include covariates. However, problems in the data collection or the linking of two different datasets often leads to a high amount of missing data. The usual approach to handle missing data results in reduced power, because phenotypic and covariate data of a twin with at least one missing value cannot be used for statistical inference.

In this paper, we present a full information approach that entails modelling covariance of all data, both phenotypic data as well as covariate data. The covariance structure for the covariates in a twin pair was decomposed into between and within family covariance. This makes it possible, to use *all* available data, which makes the new approach more powerful than the usual approach.

In a simulation study, the performance of the full information approach was compared to the usual approach under different conditions. Independent of missingness scenario (MCAR, MAR and MNAR), amount of missingness (2, 6 or 10 % of the total number of covariate values) and number of covariates (two, three, four and five), standard errors for all variance components were lower for the new approach than for the usual approach. Furthermore, standard errors of the full information approach were constant while the power of the usual approach rapidly decreased with increasing number of covariates. Note that this pattern has to do with the fact that, in the usual approach, twins with at least one missing value do not enter the analysis. Therefore, although the percentage of missingness remained constant, the probability that a twin ends up with at least one missing value was higher with increasing number of covariates. This reduced the number of twins that entered the analysis, resulting in decreasing power with increasing number of covariates.

Note that we used the same covariance structure for MZ and DZ twin pairs to model covariate covariance between families (i.e., ${\mathrm {\Sigma _b}}$) and covariance within families (i.e., ${\mathrm {\Sigma _w}}$). Therefore, the exact results of the simulation study are restricted to this situation. But there is nothing against specifying different covariance structures for MZ and DZ twin pairs. Simulating and analysing covariate data with different covariance structures among MZ and DZ twins might lead to slightly different effects on power, as the probability that covariate data are missing in both twins might be different for MZ and DZ twins respectively. Generally though the effects on power will be the same: the full information approach is more powerful than the usual approach. Depending on the particular application, it might be more appropriate to use different covariance structures for MZ and DZ twins when differences in the covariance structure are expected a priori or when the data seem to suggest it (i.e. twin correlations on the covariate data might be higher in MZ twin pairs than in DZ twin pairs).

To illustrate the new approach, the effects of specific covariates on the test scores of 990 12-year-old twin pairs on a national Dutch educational achievement test (*Eindtoets Basisonderwijs*) in primary school were investigated. We used school-aggregated measures (school denomination, pedagogical philosophy, school size) and the sex of a twin as covariates. Similar to earlier findings on *Eindtoets Basisonderwijs* scores of a Dutch sample (Bartels et al. 2002), the results suggest that differences in test scores are mainly due to genetic influences. There was no covariate that had a significant effect on individual differences in test scores. As, however, a substantial part of the phenotypic variance could be explained by environmental variance, this suggests that there are environmental influences that were not investigated in this study that cause individual differences in *Eindtoets Basisonderwijs* test scores. Variables that might be important but were not examined in this paper might for example be resources the twins have (e.g. libraries in the neighbourhood or books at home) or the composition of their class (e.g. the IQ of their classmates). Another explanation for the non-significant result could be that environmental influences on students test scores are highly multifactorial, meaning that there are a lot of influences that each have small effects and contribute to variance in test scores when they are combined.

In the application, 18 % of the total number of covariate answers was missing (i.e., 1432/(four $\times$ 1980 individual twins)). This shows that even the most extreme missingness condition (i.e., 10 %) of the simulation study is realistic and to be expected in real data applications. When the usual approach would be applied to the same data, this would result in the loss of the phenotypic as well as covariate data of in total 496 individual twins, reducing the twin sample from 1980 individual twins to 1484 individual twins (75 % of the original sample size). This highlights the added value of the full information approach over the usual approach in practical situations.

In conclusion, as it could be shown that the full information approach is more powerful than the usual approach deletion and can be easily applied in OpenMx (Boker et al. 2011) by using the syntax we provide here, we advise researchers to use the new approach whenever (a) more than 3 % of the total covariate data are missing and (b) when more than two covariates are used in the analysis.
